# Bacterial Isolates and Antibiotic Sensitivity among Gambian Children with Severe Acute Malnutrition

**DOI:** 10.1155/2011/825123

**Published:** 2011-07-14

**Authors:** Uduak A. Okomo, Danlami Garba, Augustin E. Fombah, Ousman Secka, Usman N. A. Ikumapayi, Jacob. J. Udo, Martin O. C. Ota

**Affiliations:** ^1^Medical Research Council (UK) Laboratories, Atlantic Road, Fajara, P.O. Box 273, Banjul, Gambia; ^2^Department of Paediatrics, University of Calabar teaching Hospital, PMB 1278, Calabar, Cross River State, Nigeria

## Abstract

*Background*. Establishing the pattern of infection and antimicrobial sensitivities in the local environment is critical to rational use of antibiotics and the development of management algorithms. *Methods*. Morbidity history and physical examination of 140 children with severe acute malnutrition were recorded. Their blood, stool, and urine samples were cultured and antibiotic sensitivity patterns determined for any bacterial pathogens isolated. *Results*. Thirty-eight children had a pathogen isolated from blood culture, 60% of which were considered contaminants. Coagulase negative staphylococcus was the predominant contaminant, while the major causes of bacteraemia were nontyphoidal *Salmonella* (13%), *S. pneumoniae* (10%), and *E. coli* (8%). *E. coli* accounted for 58% of the urinary isolates. No pathogen was isolated from stool. In vitro sensitivity by disk diffusion showed that 87.5% of the isolates were sensitive to ampicillin and/or gentamicin and 84.4% (27/32) to penicillin and/or gentamicin. *Conclusions*. A combination of ampicillin and gentamicin provides adequate antibiotic cover for severely malnourished children in The Gambia.

## 1. Introduction

Severe acute malnutrition (SAM) results from a relatively short duration of nutritional deficit that is often complicated by marked anorexia and concurrent infective illness [1]. Globally, comorbidities such as diarrhoea, pneumonia, and malaria, which result from a relatively defective immune status, remain the major causes of death among children with SAM [2]. Children with complications require hospital care due to the attendant high risk of mortality [3]. The high prevalence of bacteraemia, urinary tract infections, diarrhea, and pneumonia among children with severe malnutrition [4–7] coupled with an atypical clinical presentation of sepsis justifies the routine use of empirical antibiotic treatment in the initial phase of inpatient management as recommended by WHO [8, 9]. However, the choice of antibiotics has to be guided by locally prevalent pathogens and their antibiotic susceptibility patterns. There are few local studies on the spectrum of bacterial isolates affecting malnourished children and their antibiotic sensitivity since the HIV pandemic [10]. The objectives of this study were to evaluate the prevalence of acute bacterial infections and their antibiotic sensitivity in children aged 6–59 months with SAM admitted to the paediatric ward of the Medical Research Council (MRC) Unit's hospital, Fajara, The Gambia.

## 2. Methods

### 2.1. Study Design and Participants

In this prospective study, children with SAM who were severely ill (apathetic or irritable), with poor appetite, or who had medical complications (hypothermia, hypoglycaemia, broken skin, and respiratory or suspected urinary tract infection), admitted into the paediatric ward of the MRC Unit's hospital, and who fulfilled the inclusion and none of the exclusion criteria, were consecutively recruited into the study. SAM was defined as a very low weight for height (below −3z scores of the median NCHS/WHO growth standards), by visible severe wasting, or by the presence of nutritional oedema [8]. Children with nonnutritional causes of oedema, a known disorder, congenital malformation, or chronic infection that could lead to malnutrition and those who routinely take antibiotics/had taken any antibiotics within the two weeks prior to presentation were excluded from the study. Children with known HIV infection and therefore receiving cotrimoxazole prophylaxis were also excluded. Written informed consent was obtained from caregivers of children before enrolment. The study was approved by the Joint Gambia Government-MRC Unit, The Gambia Ethics Committee.

A standardized clinical form was used to collect sociodemographic information, clinical symptoms and their duration, immunization history, anthropometric measurements, physical signs, results of laboratory investigations, and the patient's final outcome. Children received routine medical care as indicated in the WHO guidelines for the management of complicated SAM which included parenteral antibiotics, after relevant body fluid samples were taken for isolation of bacterial pathogens [8]. Caregivers received pretest counselling for HIV testing by a trained counselor. Where consent was given, an HIV serologic test was performed; a positive result for children ≥18 months of age was confirmed with a second serologic test, while real-time polymerase chain reaction (RT-PCR) was performed to confirm a positive serology result for those <18 months of age. Caregivers of children who tested positive for HIV were counselled and referred to the MRC Unit's HIV clinic for further management and followup.

### 2.2. Sample Collection

All children had blood samples collected for culture at admission, prior to antibiotic administration. Blood was obtained for culture by venepuncture after the intact skin was cleansed with 70% ethyl alcohol. Additional samples taken for culture included swabs from discharging ears or ulcerative skin lesions and fresh stool from children with diarrhoea. The choice of method for urine sample collection depended on the age of the child and the fullness of the bladder at the time of examination. For children <12 months of age, urine samples were collected by percutaneous suprapubic aspiration of the bladder (SPA). In the event of a “dry tap” and for older children, urine was collected by urethral catheterization or collection of a freshly voided “clean-catch” specimen. For SPA, the overlying skin was first cleaned with a cotton wool pad soaked in 70% ethyl alcohol. Where catheterization was performed, the external genitalia were cleansed. All blood and, whenever possible, urine and stool specimens were collected from each child before the commencement of antibiotics.

### 2.3. Bacteriologic Methods

Blood samples for culture were inoculated into commercially produced vials containing BD BACTEC Peds Plus/F media (enriched Soybean-Casein Digest broth with CO_2_) following manufacturer's instructions for quality control and blood volume requirements. This medium supports the growth of antibiotic-exposed organisms thereby enhancing the recovery of susceptible isolates. Vials inoculated with blood samples were incubated in the automated BACTEC 9050 blood-culture system (Becton Dickinson, Temse, Belgium). When there was a positive signal from the machine, an aliquot was obtained from the vial with syringe and needle to be further examined by Gram stain and subcultured onto appropriate solid media. Negative vials were also checked by Gram stain and subcultured prior to discarding as negative. Subcultures were performed twice onto blood agar, chocolate agar, and MacConkey agar plates. Plates were incubated for 18–24 hours as follows: blood agar plates—aerobically and anaerobically at 37°C; chocolate agar in 5% CO_2_ at 37°C; MacConkey agar—aerobically at 37°C. Plates were examined for pathogens using standard procedures. Blood cultures were considered positive if a definite pathogen (e.g., *S. pneumoniae*, *H. influenzae*, *Streptococcus pyogenes*, *Salmonella* species.) was isolated. Coagulase-negative staphylococci, Micrococcus species, Bacillus species, and isolates with scanty growth not on the line of inoculum that failed to grow on subculture were regarded as contaminants.

All urine samples were examined microscopically and plated onto cysteine lactose electrolyte deficient (CLED) agar using a standardized loop. Plates were incubated aerobically at 37°C overnight and examined for growth the following morning. The method of urine sample collection was indicated on the laboratory request form, and this influenced the interpretation of the culture findings. The diagnosis of bacteriuria was based on the finding of any bacterial growth in urine obtained by SPA or >10^5^ colonies/mL of urine obtained from a freshly voided specimen.

Stool specimens were examined microscopically for parasites. The laboratory routinely cultures stool specimens for *Salmonellae* and *Shigellae* only. Antimicrobial sensitivity patterns of bacterial isolates from blood, urine, or stool were determined by Kirby-Bauer disk diffusion test using interpretative criteria described previously [11]. Sensitivity patterns of organisms regarded as contaminants by the microbiology laboratory such as Coagulase negative *Staphylococcus* (CONS), *Bacillus*, and *Micrococcus* species are not routinely determined. The microbiology laboratory has routine external quality assurance programme with the United Kingdom National External Quality Assessment Service.

### 2.4. Data Analysis

All data were double entered into an Access database and checked for errors. The characteristics of children with positive blood, urine, and stool cultures were compared with those with negative cultures, respectively. Persistent diarrhoea was defined as a diarrhoeal episode lasting more than 14 days. Fever was defined as an axillary temperature ≥37.5°C and hypothermia as a temperature <35°C. Tachycardia was defined as a pulse rate ≥160 beats per minute in children below 12 months of age and a rate ≥140 in children aged 12–59 months. A raised respiratory rate was ≥50 breaths per minute in children below 12 months and ≥40 in children aged 12–59 months. Wilcoxon rank sum test was used to compare continuous variables associated with positive blood and urine cultures. For categorical variables, proportions were compared using chi-squared or Fisher's exact test where appropriate. All statistical analyses were performed with STATA Version 11 (Stata Corp., College Station, Tex, USA) and statistical significance defined as *α* < 0.05 (two sided).

## 3. Results

One hundred and forty children who met the inclusion criteria were enrolled in the study between November 2007 and December 2008. The median age of the children was 19.1 months (interquartile range [IQR] 13.3–24.2 months), and 46.4% were female (Table 1). Forty five (32.1%) of those enrolled had oedema, with or without a weight-for-height below −3 SD. Chest radiographs were carried out on 101 children, and radiological evidence of pneumonia was present in 25.4% (18/71) of those with a history of cough compared with 40% (12/30) of those without. None of the children had acid fast bacilli recovered from gastric washings. Of the 91 children who received a tuberculin skin test (Mantoux), only two had a positive reaction, one of whom had radiological lung consolidation; both children, however, received treatment for tuberculosis. There were 38 positive blood cultures all of which grew bacterial pathogens. Urine samples were obtained from 97 children before antibiotics were commenced; 16 (16.5%) of the samples were positive for a bacterial pathogen. Stool samples were collected from 54 children at admission, 43 (79.6%) of whom had a history of diarrhoea. One child had cysts of *Entamoeba coli* and another *Strongyloides stercoralis* in the stool; there were no bacterial positive stool cultures. HIV-1 infection was present in 27 (28.7%) of the 94 children tested for HIV. Eight (5.7%) children died in hospital; three of the deaths occurred within the first 48 hours of admission. Only one of the children that died was HIV positive; this child also had a positive blood culture which grew *S. pneumoniae. * Two other children who died also had positive blood cultures which grew *E.coli* and *Haemophilus influenzae* (nontype b), respectively.

Seventy-one percent (27/38) of all blood isolates were Gram-positive aerobes, 26.3% (10/38) were Gram-negative aerobes, and one culture had unspecified mixed bacterial growth (Table 2). The most frequent isolates were CONS in 50% (19/38), nontyphoidal *Salmonellae* (NTS) in 13.2% (5/38), and *Streptococcus pneumoniae* in 10.5% (4/38) of cultures. Only 15 of the isolates were considered to be a genuine pathogen giving an overall prevalence of bacteraemia of 10.7%. The 23 children whose blood cultures yielded contaminants were compared to those whose cultures were negative; there were no significant differences between the two groups with regards to all the clinical parameters. Thus, those with contaminants were excluded from further analyses. In the univariate analysis, a history of cough, persistent diarrhoea, vomiting, splenomegaly at presentation, or a haemoglobin level <8 g/dL were found to be significantly associated with bacteraemia (Table 3). However, in the multivariate model fitted using all factors with a *P* value less than 0.1 in the univariate analysis, the odds of bacteraemia in children with a history of vomiting and anaemia at presentation were five and three times greater, respectively, relative to their comparison groups although these findings were of borderline statistical significance (Table 3). Seven children with bacteraemia had anaemia at presentation; 3/7 (42.8%) with *Streptococcus pneumoniae *bacteraemia, 2/7 (28.6%) with *E.coli*, and 1/7 (14.3%) each with *Haemophilus influenzae* (nontype b) and NTS bacteraemia, respectively.

Bacteraemia was present in 11.1%, 8.9%, and 13% of children who were HIV positive, HIV negative, and those not tested, respectively. There was no significant difference in the proportion of contaminated cultures between children who were found HIV positive, HIV negative, and those not tested (*P* = 0.675).

There were a total of 17 urinary isolates (Table 2) giving a prevalence of bacteriuria of 16.5%. The distribution of urine cultures by method of sample collection is shown in Table 5. *E. coli* accounted for 55.6% of the isolates; one child had polymicrobial bacteriuria (*Proteus mirabilis* and *Providentia alcalifaciens*). Children with or without bacteriuria did not differ significantly with regard to symptoms and other clinical parameters. Four (25%) of the children with bacteriuria had concomitant bacteraemia; one child had the same organism *E. coli*, cultured from both sites.

All four Isolates of *S. pneumoniae* were susceptible to penicillin, ampicillin, and chloramphenicol, (Table 4) while only one was susceptible to ceftriaxone. All 13 isolates of *E. coli* were susceptible to gentamicin, nitrofurantoin, ciprofloxacin, cefuroxime, cefotaxime, and ceftriaxone, but only 77% were susceptible to chloramphenicol. All NTS isolates were 100% susceptible to cotrimoxazole, gentamicin, chloramphenicol, ciprofloxacin, cefuroxime, and cefotaxime; with 80% of them being sensitive to ampicillin. Eight-seven percent (28/32) of all the bacterial isolates demonstrated sensitivity to ampicillin and/or gentamicin, while 84.4% (27/32) isolates were sensitive to penicillin and/or gentamicin. Seventy-eight percent (25/32) of the organisms, respectively, were sensitive to ampicillin and/or chloramphenicol or penicillin and/or chloramphenicol.

## 4. Discussion

We have studied bacterial isolates and antimicrobial sensitivity among 140 children aged 6–59 months of age with SAM admitted to the paediatric ward of MRC Unit's hospital, The Gambia. 

The prevalence of bacteraemia among children with severe malnutrition in this study of 10.7% is similar to that reported across Sub-Saharan Africa in which the prevalence of bacteraemia ranged from 8.6% to 70% in West Africa; [10, 12] 9.2% to 36% in East Africa [13–17], and 7.7% to 13% in South Africa [4, 6, 18]. Causative agents of bacteraemia, however, vary geographically with most studies reporting a predominance of Gram-negative enteric bacteria (GNEB) [13, 14, 16–19] and a few in which Gram-positive aerobes predominated, mostly *Staphylococcus species* [4, 10, 12, 20–22]. In our study, GNEB accounted for 53% of the bacteraemic episodes, with nontyphoidal *Salmonella* (NTS) being the most common isolate. Surprisingly, *Staphylococcus aureus* was not among any of the isolates recovered from blood despite being isolated from 85% of cases of skin sepsis. All isolates of *H. influenzae* found among the children in this study were nonserotypable, and this could be explained by the nearly complete coverage of* H. influenzae* type b (Hib) vaccine in The Gambia [23].

Though considered a contaminant, coagulase-negative staphylococcus (CONS) was the predominant blood isolate in this study accounting for 50% of isolates (Table 2). Studies from several different regions have reported CONS rates in blood cultures ranging from 26.7 to 40% [16, 22, 24] although the high CONS isolation rate in this study may have been due to the use of 70% alcohol alone to clean the skin prior to venepuncture, rather than using it in combination with 10% povidone iodine as in other studies [16, 18, 22]. However, CONS are prominent components of the microbial skin flora, and any interruption in the normal skin defence barrier as may occur in severe malnutrition facilitates entry of these organisms into the bloodstream with resultant bacteraemia [22]. Moreover, CONS are well recognized as a significant cause of sepsis among critically ill and immunosuppressed children [25, 26]. Thus, judging the clinical significance of CONS is vital, but a clinical dilemma that could have a profound impact on an institution's bloodstream infection rates [27]. The proportion of CONS isolates from positive blood culture samples in our laboratory was 45.3% and 42.7% in 2006 and 2007, respectively, (personal communication from laboratory manager); though these figures include samples from adults and children in the ward, they are similar to the 50% observed in this study and support the view that the CONS isolates in this study were truly contaminants. Antibiotic sensitivity patterns were not performed for CONS in this study and have been found to be of little benefit in differentiating genuine CONS bacteraemia from contamination [28]. Although some authors recommend that empirical therapy with CONS-sensitive antimicrobials particularly vancomycin be given to critically ill children, there is a need for the practice of proper hand-washing techniques and venepuncture by medical staff as well as careful evaluation of CONS isolates from blood cultures before instituting therapy so as to avoid unnecessary use of antibiotics and the consequent risk of local antibiotic resistance [29]. A history of vomiting and anaemia were both found to be associated with an increased risk of bacteraemia in this study though this finding was of borderline statistical significance. Bacteraemia due to NTS bacteraemia is known to be associated with an increased risk of anaemia in African children [30, 31]; however, among the children with bacteraemia and anaemia in our study, *Streptococcus pneumoniae* was the predominant isolate. While this finding should be interpreted with caution on account of the small number of children with concomitant bacteraemia and anaemia in this study, it is expected that there will be a steady decline in the incidence of invasive pneumococcal disease with the introduction of the pneumococcal conjugate vaccine in the EPI schedule in The Gambia. HIV infection did not present any additional risk for bacteraemia among children in this study. 

The prevalence of bacteriuria of 16.5% reported in our study is consistent with most other studies in which urine isolation rates varied between 5% and 35% [4, 7, 12, 16, 17, 32–35]. The finding of *Escherichia coli* and *Klebsiella* species as the predominant urinary pathogens is also consistent with reports from previous studies where were these pathogens accounted for up to 62.5% and 12.5% of urine isolates respectively [33–36].

Even though stool samples in this study were selectively cultured for only *Salmonella* and *Shigella*, the absence of positive stool cultures compared to 15.3% in previous study more than 20 years ago [37] is surprising but could be a reflection of changes with time. 

Radiological evidence of pneumonia was found in a higher proportion of children without history of cough than those with cough. This highlights the fact that these malnourished children often have atypical presentations as a result of altered immune status and energy balance. Isolates recovered from blood may not always represent the aetiological agents of pneumonia; consequently, the antibiotic required may differ from those indicated by blood culture [38]. Lung aspirations were not done as routine clinical care in our setting; this could have increased the proportion of SAM with documented bacterial isolates [39].

Acquired bacterial resistance to first-line broad-spectrum antibiotics is increasingly common and is a significant cause of mortality [40, 41]. In most countries, there is dearth of information on the pattern of antibiotic resistance of different pathogens, and there are very little resources to obtain reliable susceptibility data on which rational treatments can be based. WHO recommends the use of a combination of intravenous ampicillin and once daily intravenous or intramuscular gentamicin. Fortunately, data from this study shows that 87.5% (28/32) of the isolates will be covered by the WHO recommended antibiotic regimen. Our data are limited by being from only one hospital and the use of in vitro susceptibility testing. The high (77%) susceptibility of *E. coli*, a major cause of both bacteraemia and bacteriuria in this study, to chloramphenicol is of benefit as it is easily absorbed and very effective when administered orally particularly when intravenous/intramuscular access is difficult.

## 5. Conclusion

Our findings in this study indicate that a combination of ampicillin and gentamicin provides adequate antibiotic cover for children with severe acute malnutrition in The Gambia. Establishing the pattern of infection and antimicrobial sensitivities in the local environment is highly recommended and critical, not only as part of infection control but also to revise policies aimed at the rational use of antibiotics and the development of management algorithms.

## Figures and Tables

**Table 1:** Characteristics of 140 children aged 6–59 months with severe acute malnutrition admitted to the paediatric ward MRC hospital, The Gambia, January 2007 and November 2008.

Characteristic	Participants^a^ (*n* = 140)
Female	65 (46.4%)
Median (IQR) age (months)	19.1 (13.3–24.2)
<12 months	27 (19.3%)
≥12 months	113 (80.7%)
Died	8 (5.7%)
History of cough	90 (64.3%)
Chronic cough	15/85 (17.7%)
History of diarrhea	97 (69.3%)
Persistent diarrhea	37/94 (39.4%)
History of fever	128 (91.4%)
History of vomiting	85 (60.7%)
Dyspnoea	16 (11.4%)
Hepatomegaly	62 (44.3%)
Splenomegaly	5 (3.6%)
Anaemia (Hb <8 g/dL)	31 (22.1%)
HIV positive	27/94 (28.7%)
Malaria parasitaemia	5/127 (3.9%)
Positive blood culture	38 (27.1%)
Positive urine culture	16/97 (16.5%)
Tachycardia^b^	26/136 (19.1%)
Tachypnoea^c^	28/131 (21.4%)
Axillary temperature ≥37.5°C	32/137 (23.4%)
White blood cell count <4 × 10^9^/L	3(2.2%)
White blood cell count ≥11 × 10^9^/L	83 (60.6%)

^a^Denominators less than 140 indicate missing data.

^b^Pulse rate ≥160 beats per minute in children below 12 months of age and a rate ≥140 in children aged 12–59 months.

^c^Respiratory rate ≥50 breaths per minute in children below 12 months and ≥40 in children aged 12–59 months.

**Table 2:** Isolated bacterial pathogens and sites of infection.

Bacterial isolates	Site of isolated pathogens				Total
	Blood	Urine^a^	Skin^b,c^	Ear^d^	
Gram positive					
*Staphylococcus aureus *			4	2	**6**
*Coagulase negative staphylococci *	19				**19**
*Streptococcus pneumonia *	4				**4**
Group A *Streptococci *			1		**1**
Group F *Streptococci *	1				**1**
*Other Streptococci *				1	**1**
*Bacillus* species	2				**2 **
Gram negative					
*Nontyphoidal salmonellae *	5				**5**
*Haemophilus influenzae* (nontype b)	2				**2**
*Escherichia coli *	3	10			**13**
*Klebsiella pneumonia *		1	1		**2**
*Klebsiella* species		2	1	1	**4**
*Micrococci *	1				**1**
*Enterobacter cloacae *		1			**1**
*Proteus* species		1	3	5	**9**
*Providencia alkali *		1			**1**
*Pseudomonas aeruginosa *		1	1	3	**5**
*Other Pseudomonas* species				1	**1**
Unspecified	1				**1 **

Total	**38**	**17**	**11**	**13**	**79**

^a^One child had more than one organism isolated from the urine.

^b^Patients with skin isolates had extensive weeping dermatosis.

^c^Four of the seven children with skin isolates had polymicrobial infection.

^d^Six of the eight children with ear isolates had polymicrobial infection.

**Table 3:** Univariate and multivariate analysis of factors associated with bacteraemia.

Characteristic	Univariate analysis		Multivariate analysis	
	OR for bacteraemia (95% CI)	*P* ^ a^	Adjusted OR for bacteraemia^b^ (95% CI)	*P* ^ a^
Sex: (*n* = 117)				
Male 63 (53.8%)	1.0		1.0	
Female 54 (46.2%)	2.6 (0.84, 8.27)	*0.09*	1.67	*0.35*
		*6*		*0*
Age: (*n* = 117)				
<12 months 22 (18.8%)	1.09 (0.28, 4.25)	*0.89*	1.0	
≥12 months 95 (81.2%)	1.0	*9*	1.05 (0.27, 4.05)	*0.94*
				*6*
History of cough: (*n *= 117)				
Yes 106 (90.6%)	10.62 (1.34, 83.9)	*0.02*	1.80 (0.52, 6.18)	*0.35*
No 11 (9.4%)	1.0	*5*	1.0	*1*
				
Chronic cough: (*n *= 70)				
≤30 days 59 (84.3%)	1.0			
>30 days 11 (15.7%)	2.8 (0.86, 11.4)	*0.15*	—	
		*1*		
History of diarrhoea: (*n* = 117)				
Yes 81 (69.2%)	0.87 (0.28, 2.77)	*0.81*	—	
No 36 (30.8%)	1.0	*8*		
Persistent diarrhoea: (*n* = 80)				
Yes 31 (38.7%)	4.5 (1.06, 18.87)	*0.04*	2.21 (0.75, 6.53)	*0.15*
No 49 (61.3%)	1.0	*1*	1.0	*1*
History of vomiting: (*n* = 117)				
Yes 70 (59.8%)	5.13 (1.1, 23.92)	*0.03*	5.78 (0.98, 33.99)	*0.05*
No 47 (40.2%)	1.0	*7*	1.0	*2*
Hepatomegaly: (*n* = 117)				
Yes 51 (43.6%)	2.98 (0.95, 9.34)	*0.06*	0.78 (0.25, 2.36)	*0.65*
No 66 (56.4%)	1.0	*2*	1.0	*6*
Splenomegaly: (*n* = 117)				
Yes 5 (4.3%)	12.5 (1.89, 82.47)	*0.00*	3.62 (0.25, 52.48)	*0.34*
No 112 (95.7%)	1.0	*9*	1.0	*5*
Anaemia: [Hb < 8 g/dL] (*n* = 117)				
Yes 25 (21.4%)	4.08 (1.31, 12.70)	*0.01*	3.41 (0.92, 12.64)	*0.06*
No 92 (78.6%)	1.0	*5*		*6*
HIV positive: (*n* = 81)				
Yes 24 (29.6%)	1.21 (0.28, 5.31)	*0.79*	—	
No 57 (70.4%)	1.0	*7*		
Malaria parasitaemia (*n* = 108)				
Yes 4 (3.7%)	2.56 (0.25, 26.58)	*0.43*	—	
No 104 (96.3%)		*2*		
Axillary temperature ≥ 37.5°C (*n* = 115)				
Yes 31 (27%)	1.42 (0.44, 4.55)	*0.55*	—	
No 84 (73%)	1.0	*2*		
White blood cell count ≥11 × 10^9^/L				
Yes 46 (40%)	0.81 (0.25, 2.60)	*0.72*	—	
No 69 (60%)	1.0	*7*		

^a^Chi-squared or Fisher's exact test.

^b^Adjusted for all factors with a *P* value <0.1 in the univariate analysis.

**Table 4:** Antibiotic sensitivity of the major blood and urine pathogens in relation to the number tested.

	Organism number susceptible/number tested		
	*S. pneumoniae*	*E. coli*	Nontyphoidal salmonellae^d^
Penicillin	4/4	NT	NT
Ampicillin	4/4	0/13	4/5
Cotrimoxazole	NT	0/13	4/4
Genticin	NT	13/13	5/5
Chloramphenicol	4/4	10/13	5/5
Nitrofurantoin	NT	8/8	NT
Ciprofloxacin	NT	13/13	5/5
Cefuroxime	NT	13/13	5/5
Cefotaxime	NT	12/12	4/4
Ceftriaxone	1/4	1/1	NT

**Table 5:** Distribution of urine culture results by method of collection.

Method of collection	Culture result		Total
	Positive	Negative^d^	
Suprapubic aspiration	5	13	18
Catheterization	10	26	36
Cleancatch	1	42	43

Total	**16**	**81**	**97**
